# Some *Brassicaceae* Extracts as Potential Antioxidants and Green Corrosion Inhibitors

**DOI:** 10.3390/ma16082967

**Published:** 2023-04-08

**Authors:** Ioana Maria Carmen Ienașcu, Adina Căta, Adriana Aurelia Chis, Mariana Nela Ştefănuț, Paula Sfîrloagă, Gerlinde Rusu, Adina Frum, Anca Maria Arseniu, Claudiu Morgovan, Luca Liviu Rus, Carmen Maximiliana Dobrea

**Affiliations:** 1 National Institute of Research and Development for Electrochemistry and Condensed Matter, 144 Dr. A. P. Podeanu, 300569 Timisoara, Romania; 2 Department of Pharmaceutical Sciences, Faculty of Pharmacy, “Vasile Goldiș” Western University of Arad, 86 Liviu Rebreanu, 310045 Arad, Romania; 3 Preclinical Department, Faculty of Medicine, “Lucian Blaga” University of Sibiu, 550169 Sibiu, Romania; 4 Faculty of Industrial Chemistry and Environmental Engineering, Politehnica University of Timisoara, 6 C. Telbisz, 300001 Timisoara, Romania

**Keywords:** *Brassicaceae* extracts, antioxidant activity, total phenolics, green corrosion inhibitors

## Abstract

Glucosinolates-rich extracts of some *Brassicaceae* sources, such as broccoli, cabbage, black radish, rapeseed, and cauliflower, were obtained using an eco-friendly extraction method, in a microwave field, with 70% ethanol, and evaluated in order to establish their in vitro antioxidant activities and anticorrosion effects on steel material. The DPPH method and Folin-Ciocâlteu assay proved good antioxidant activity (remaining DPPH, 9.54–22.03%) and the content of total phenolics between 1008–1713 mg GAE/L for all tested extracts. The electrochemical measurements in 0.5 M H_2_SO_4_ showed that the extracts act as mixed-type inhibitors proving their ability to inhibit corrosion in a concentration-dependent manner, with a remarkable inhibition efficiency (92.05–98.33%) achieved for concentrated extracts of broccoli, cauliflower, and black radish. The weight loss experiments revealed that the inhibition efficiency decreased with an increase in temperature and time of exposure. The apparent activation energies, enthalpies, and entropies of the dissolution process were determined and discussed, and an inhibition mechanism was proposed. An SEM/EDX surface examination shows that the compounds from extracts may attach to the steel surface and produce a barrier layer. Meanwhile, the FT-IR spectra confirm bond formation between functional groups and the steel substrate.

## 1. Introduction

Due to their exceptional mechanical and electrical properties, metals are commonly used in human activities [1]. The corrosion process is perhaps the most common phenomenon that causes the deterioration of metals and it is due to the electrochemical interaction of metals with the corrosive environment [2,3]. Among metals, steel is usually used in many industries due to its excellent mechanical properties. Thus, finding solutions to reduce the degradation of steel by corrosion represents a high-priority matter [4].

In order to fight against corrosion, some different strategies such as design, materials selection, electrochemical protection, coatings, and the use of inhibitors were applied. The latter is considered the easiest to apply and the most cost effective [5]. Corrosion inhibitors are chemicals that are added to metal surfaces or to the aggressive medium, reducing the rate of metals dissolution. The common corrosion inhibitors, mainly chromates and their derivatives, have proven to be dangerous substances for human life and the environment [6]. Recent approaches showed the potential of plant extracts as corrosion inhibitors [7,8,9,10], so the replacement of the traditional toxic corrosion inhibitors can be achieved.

Plant extracts contain phytochemical compounds with similar characteristics to organic corrosion inhibitors and show advantages such as low cost, wide availability, nontoxicity, biodegradability, and biocompatibility, which recommend them as an eco-friendly alternative to classic corrosion inhibitors [9,11].

Crops of cabbage, acclimatized broccoli, black radishes, rape, and cauliflower are cultivated in Romania and are very cheap raw materials. Natural compounds of different species of the *Brassicaceae* family were easily extracted [12,13,14,15]. Plants from the *Brassicaceae* species contain several phytochemical compounds such as glucosinolates, glucosides, phenolic acids, erucic acid, polyphenols and tocopherols, carotenoids, flavonoids, alkaloids, terpenoids and terpenes, phytoalexins, and phytosteroids [16]. Thus, the extracts of such plants are used in the food industry, pharmaceutical industry, and alternative medicine [17,18,19,20,21,22] due to their diverse biological activities, mainly antimicrobial [19,20,21,22] and antioxidant effects [14,15,19].

Several extracts from some *Brassicaceae* species demonstrated excellent inhibition effects on steel corrosion [23,24,25]. Hence, an aqueous extract of *Brassica oleracea* was proven to retard Q235 steel corrosion in two harsh acid environments (0.5 M H_2_SO_4_ and 1 M HCl) [25]. The inhibition efficiency of *Brassica oleracea* extract (99% ethanol) on the corrosion of pipeline steel in 0.5 M H_2_SO_4_ has also been demonstrated [24]. On the other hand, some *Brassica campestris* extracts were capable of inhibiting Cor-Ten steel corrosion in HCl and NaCl solutions [23].

Given the composition of *Brassicaceae* extracts and their proven efficiency, they can successfully replace conventional toxic inhibitors and extend the possibility of “smart coatings“ by inducing a response in the coating and/or substrate to improve the inhibition of corrosion [16].

The aim of this research was to demonstrate the high antioxidant qualities and corrosion inhibition power of five indigenous *Brassicaceae* species (cabbage, broccoli, black radish, rapeseed, and cauliflower) in an aggressive 0.5 M H_2_SO_4_ environment.

## 2. Materials and Methods

### 2.1. Materials

The 1,1 diphenyl-2-picrylhydrazyl (DPPH), Folin-Ciocâlteu reagent, gallic acid (GA), 99% ethanol (analytical grade), methanol (analytical grade), and 98% sulphuric acid were all purchased from Merck (Germany). Romanian vegetables, white cabbage, broccoli, black radish, and cauliflower were purchased from a supermarket and rapeseed was collected from a local farm (Timiş county) in 2022. Ultrapure water was obtained in the lab (EASYpure RoDi—Barnstead apparatus).

### 2.2. Extraction

Plant materials were washed, air dried, chopped/ground, and freeze-dried. Then, 1 g dried material and 10 mL 70% ethanol were subjected to extraction in a microwave field (2450 MHz). Extraction was carried out at 120 °C, 15 min., in an MSW-2 Berghof oven (1000 W) equipped with a rotor with 10 Teflon vessels DAP-60K. After extraction, the solid part was removed by filtration, and the liquid phase was concentrated under a vacuum to 10 mL (extracts A).

### 2.3. Antioxidant Activity and Total Phenolics

For spectrophotometric measurements, a Jasco V530 apparatus (Abl&E-Jasco, Wien, Austria) was used. The antioxidant activities were determined using the DPPH method [26]. The calibration curve used was A = 11048 · C_DPPH_ + 0.0037 (R^2^ = 0.999). At 2.9 mL methanolic solution of DPPH (~9.5 × 10^−5^ mol/L), 0.1 mL ethanolic extract A was added and the change of DPPH color was from mauve to yellow, caused by the consumption of DPPH radicals by the existing antioxidants compounds, was followed (λ = 515 nm). The remaining DPPH was calculated with the following equation:

(1) $$\mathrm{Remaining}\;\mathrm{DPPH}\;\left(\%\right)=\frac{C_{\mathrm{DPPH}\left(t\right)}}{C_{\mathrm{DPPH}\left(t=0\right)}}\times 100$$

where C_DPPH(t)_ is the value of the DPPH concentration in the presence of the extract at time t and C_DPPH(t=0)_ at time 0.

The content of total phenolics was determined by the Folin-Ciocâlteu method, as described by Ștefănuț et al. [26]. Gallic acid was used as the reference compound and the total phenolic content was expressed as mg GAE/L.

### 2.4. Electrochemical Experiments

The electrochemical tests were carried out with 0.5 M H_2_SO_4_, in a conventional glass three-electrode cell with a Pt counter electrode, a saturated calomel (SCE) as reference electrode, and a working electrode (WE) made from a steel disk. The WE was embedded in a Teflon jacket by screwing. The exposed area was A = 0.28 cm^2^. Before use, the WE was gradually polished with emery paper (1000–1400), cleaned with detergent and water, and finally, with acetone. Then, 30 mL 0.5 M H_2_SO_4_ plus 1 mL of each extract were used in the tests. All the experiments were carried out at an open circuit (OCP) for 30 min in order to obtain a stable potential at room temperature. Electrochemical tests were performed with a Voltalab 80 (Radiometer, Denmark). The potentiodynamic measurements were started at −600 mV cathodic potential to anodic potential +250 mV, at a scan rate 1 mV/s. The data were registered and analyzed using VoltaMaster4 software. Parameters such as corrosion potential *E_corr_*, corrosion resistance *Rp*, Tafel slopes (*βa*, *βc*), *β* corrosion intensity *I_corr_*, and corrosion rate, *v_corr_*, were obtained by the Tafel extrapolation method.

The inhibition efficiency was defined by Equation (2).

(2) $$\mathrm{IE}\left(\%\right)=\left(\frac{v_{corr}^{0}-v_{corr}}{v_{corr}^{0}}\right)\times 100$$

where: *v^0^_corr_* and *v_corr_* are corrosion rates in the absence of and in the presence of different extracts, respectively.

### 2.5. Weight-Loss Experiments

Similar steel disks (same composition and dimensions) as those used in the electrochemical experiments were polished with different grades of emery paper (1000–1400 mesh), washed with ultrapure water, degreased with acetone, and air dried. The specimens were immersed in the corrosion medium (15 mL 0.5 M H_2_SO_4_) and kept for 24 h at 20, 40, 50, and 60 °C, in the absence and the presence of 0.5 mL of extract A. Finally, the steel disks were removed, rinsed with water and acetone, dried in warm air, and stored in a desiccator. Weight loss was determined by gravimetric measurements using an analytical balance with a precision of 0.1 mg.

The corrosion rate (*v*_corr_, mm/year) of steel in 0.5 M H_2_SO_4_ with and without extract A was calculated with Formula (3) [27]:

(3) $$v_{\mathrm{corr}}=\frac{87.6\cdot \Delta W}{S\cdot t\cdot D}$$

where ΔW is the corrosion weight loss of the steel specimen (mg), S is the area of the steel specimen (cm^2^), t is the exposure time (h), and D the density of steel (g/cm^3^).

The inhibition efficiency was obtained using Equation (2), using for calculation the values of corrosion rate (*v*_corr_) obtained by the gravimetric method.

The temperature effect on the corrosion rate of steel in 0.5 M H_2_SO_4_ was studied. These tests were executed in the absence and presence of 0.5 mL extracts A for 24 h, at 20, 40, 50, and 60 °C. The relationship between the corrosion rate (*v_corr_*) of steel in an acidic media and temperature (T) is expressed by the Arrhenius equation [28]:

(4) $$v_{\mathrm{corr}}=A\cdot e^{-E_{a}/\mathrm{RT}}$$

where *v_corr_* is the corrosion rate, A is the Arrhenius pre-exponential factor, E_a_ the apparent activation energy for corrosion process, R is the universal gas constant, and T the absolute temperature.

The values of enthalpy of activation (ΔH*) and entropy of activation (ΔS*) were calculated using an alternative form of the Arrhenius equation [27]:

(5) $$v_{\mathrm{corr}\left(G\right)}=\frac{R\;T}{N\;h}\cdot e^{\Delta S^{*}/R}\cdot {\;e}^{-\Delta H^{*}/\mathrm{RT}}$$

where h is the Planck’s constant, N is the Avogadro’s number, T is the absolute temperature, and R is the universal gas constant.

### 2.6. Scanning Electron Microscopy (SEM) and EDX Studies

After the corrosion tests, the steel disks were washed and dried, and subjected to an SEM/EDX analysis. The SEM images and the atomic content were registered using the scanning microscopy method (Scanning Electron Microscope Inspect S + EDAX Genesis XM 2i—FEI, Holland), at 30 kV, in vacuum mode, at 400–6000 magnification for all the samples.

### 2.7. FT-IR Analysis

The FT-IR spectra were recorded using a Bruker Vertex 70 spectrometer (Bruker Optik GmbH, Rosenheim, Germany) equipped with a Platinium ATR unit, Bruker Diamond A225/Q.1., at room temperature (4000–400 cm^−1^) with a nominal resolution of 4 cm^−1^ with 64 scans.

## 3. Results

**Figure 1:**
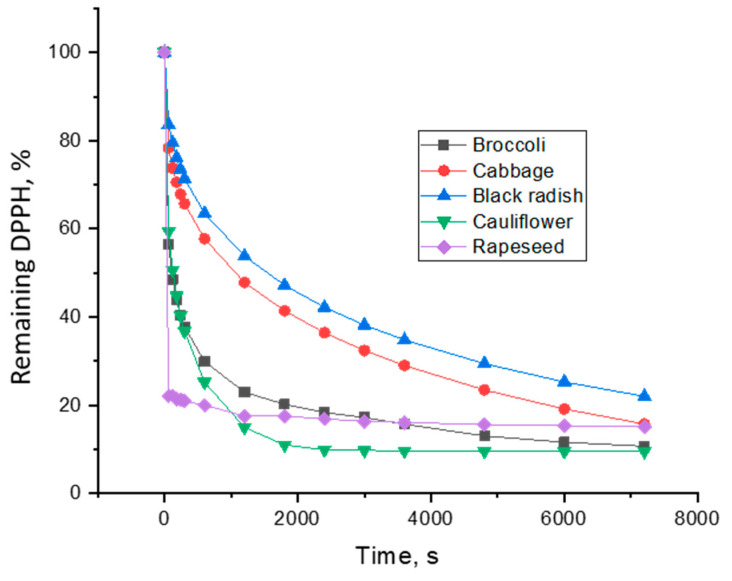
Antioxidant behavior of *Brassicaceae* extracts A.

Five extracts from Romanian cabbage, acclimatized broccoli, black radish, cauliflower, and rapeseed were obtained (extracts A) and were analyzed by the UV-Vis technique, in order to evaluate their antioxidant effect and total phenolics. Figure 1 shows the dependence of remaining DPPH (%) on time and permits the evaluation of antioxidant activities of the studied extracts. The values obtained for antioxidant activities and the total phenolic content of the extracts A are presented in Table 1.

**Figure 2:**
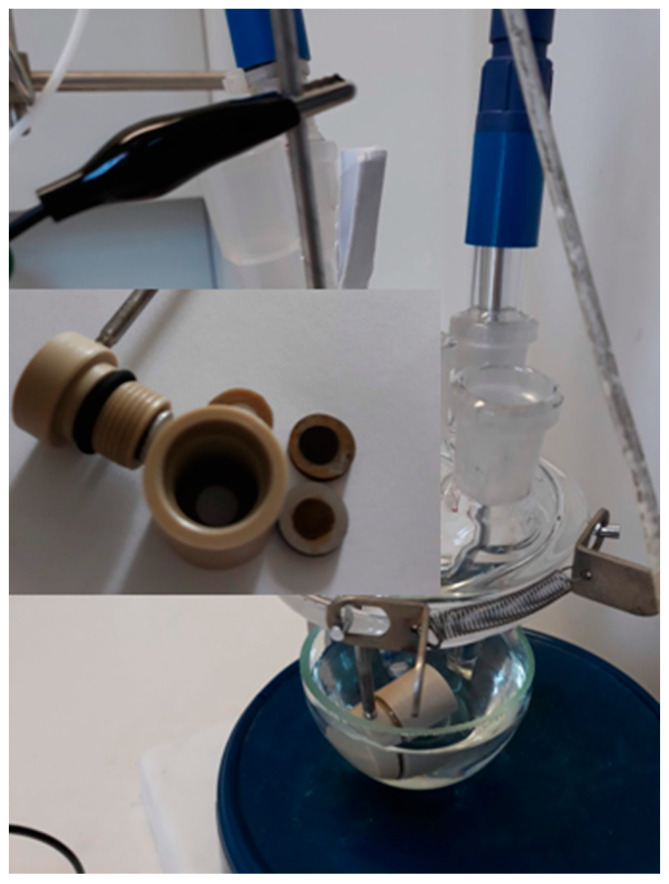
Electrochemical cell and disk work electrode.

For the electrochemical tests, two concentrations of the extracts were used, one corresponding to the extract A and one obtained by 20 times dilution of the extract A (extract B). The electrochemical behavior was evaluated using an experimental cell with a disk work electrode (Figure 2), the steel composition of the work electrode is presented in Table 2.

**Figure 3:**
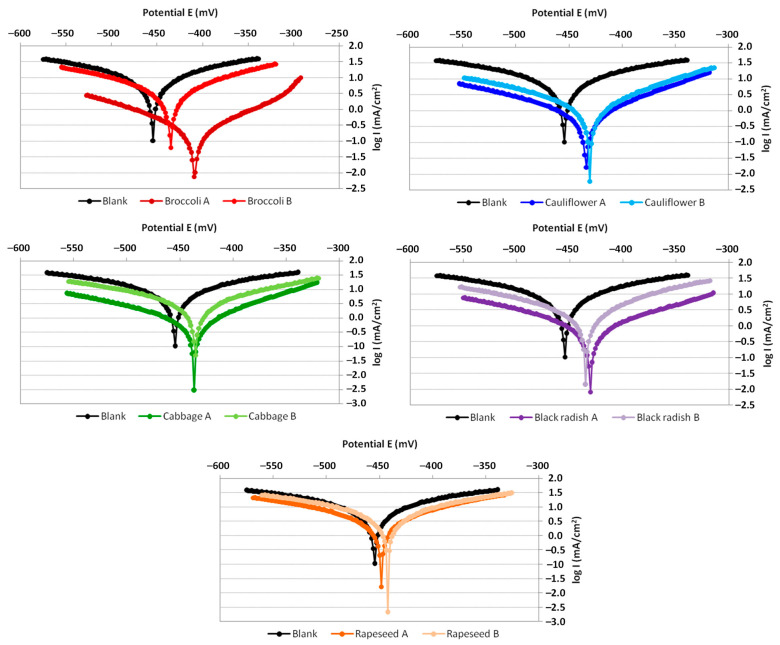
Potentiodynamic polarization curve for steel in 0.5 M sulfuric acid in the absence and the presence of *Brassicaceae* extracts A and B.

The polarization curves for steel in 0.5 M H_2_SO_4_ (blank) with and without the two tested concentrations of broccoli, cauliflower, black radish, cabbage, rapeseed extracts are presented in Figure 3.

The electrochemical parameters, corrosion potential (*E_corr_*), corrosion resistance (*Rp*), corrosion current density (*I_corr_*), cathodic Tafel slope (*βc*), anodic Tafel slope (*βa*), corrosion rate (*v_corr_*), and inhibition efficiency (*IE*), obtained from potentiodynamic polarization curves, are presented in Table 3.

**Figure 4:**
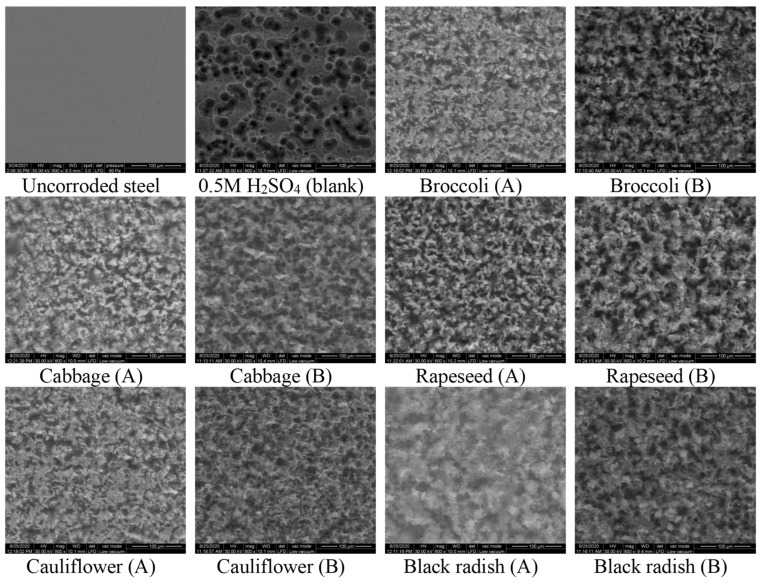
SEM images (800×) for steel, exposed to 0.5 M H_2_SO_4_ with and without *Brassicaceae* inhibitors, after electrochemical tests.

Figure 4 shows the SEM images registered for the surface of the steel specimen before and after the electrochemical experiments, with and without extracts A and B.

The results from the weight loss measurements for the corrosion of steel disks in 0.5 M H2SO4 in the absence and presence of extracts A, for 24 h, at four different temperatures, are given in Table 4.

**Figure 5:**
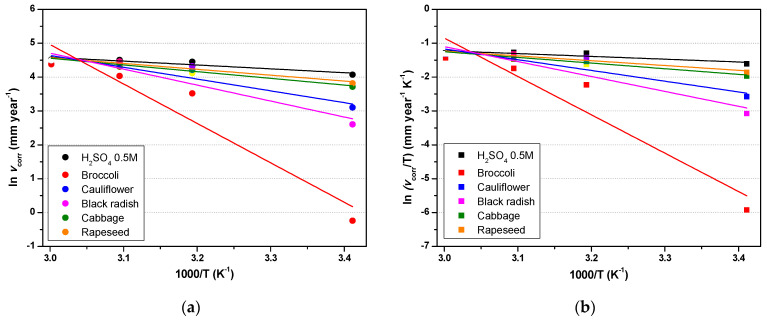
Arrhenius plots for steel in 0.5 M H_2_SO_4_ in the absence and presence of *Brassicaceae* extracts A: (**a**) ln v_corr_ vs. 1000/T; (**b**) ln(v_corr_/T) vs. 1000/T.

The apparent activation energies (Ea) were determined by linear regression between ln *v_corr_* and 1000/T (Figure 5a) and the results are shown in Table 5. Straight lines with a regression coefficient close to unity (R^2^ > 0.9) were plotted, from which the apparent activation energies (E_a_) obtained from the slope (−E_a_/2.303R) of the lines were determined.

The enthalpy of activation (ΔH*) and entropy of activation (ΔS*) for steel dissolution in 0.5 M H_2_SO_4_ with and without inhibitor extracts, were established by plotting the ln (*v_corr_*/T) against 1000/T. The straight lines plotted are illustrated in Figure 5b. The values of ΔH* and ΔS*, calculated from the slope -ΔH*/R and the intercept (ln(R/Nh) + ΔS*/R) are shown in Table 5.

**Figure 6:**
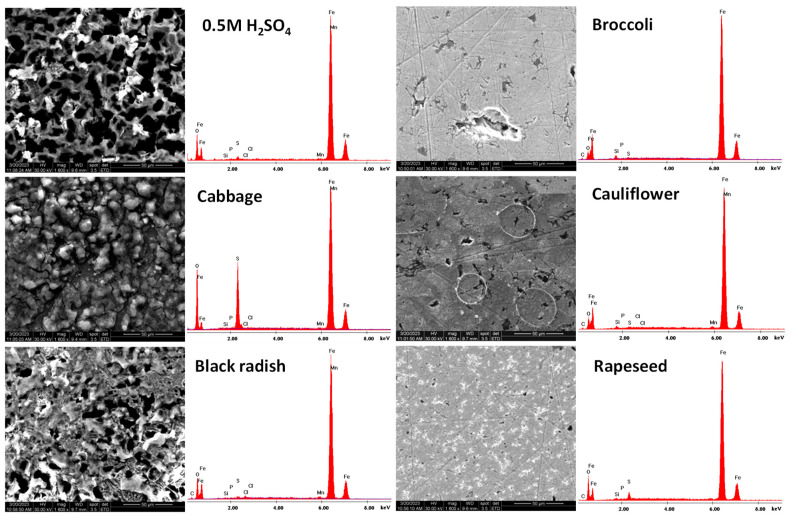
SEM images/EDX spectra of the steel surface after the weight-loss experiments, with and without extracts A.

The SEM images recorded for the steel surface after the weight loss experiments, with and without extracts A and the EDX spectra of the surface of the steel disks before and after immersion for 24 h, at 20 °C, in 0.5 M H_2_SO_4_ solution, with and without extracts A, are displayed in Figure 6 and the atomic content is presented in Table 6.

**Figure 7:**
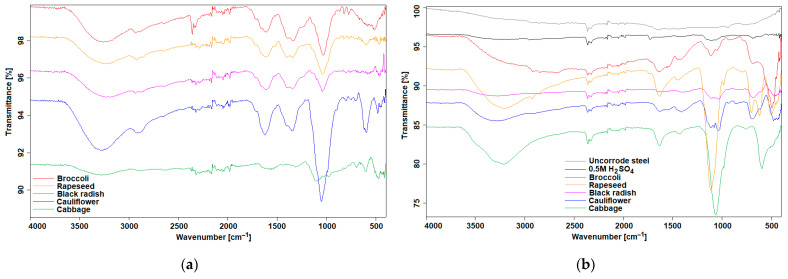
FT-IR analysis: (**a**) *Brassicaceae* extracts; (**b**) Steel surface after 24 h of exposure to a solution of 0.5 M H_2_SO_4_, with and without *Brassicaceae* extracts, at 20 °C.

FT-IR analysis (Figure 7) of the extracts and for the steel surface after 24 h of exposure to a solution of 0.5 M H_2_SO_4_, with and without *Brassicaceae* extracts, at 20 °C, was carried out. The changes in the FT-IR spectra of the inhibitor film compared to *Brassicaceae* extracts are presented in Table 7.

## 4. Discussion

According to our previous research [19], the microwave method using 70% EtOH, is a proper eco-friendly method for plant extraction. Regarding the antioxidant capacity and total phenolics of the *Brassicaceae* extracts, all analyzed extracts showed very good antioxidant activities (9.54–22.03% remaining DPPH) (Figure 1, Table 1), however, the results do not correlate with the values obtained for total phenolics (Table 1). This could be due to the presence of other compounds in the plant matrix, i.e., glucosinolates [19,29].

In a previous study, we determined the composition of the glucosinolates of the extracts using HPLC-DAD. Sinigrin was found in all extracts, being the predominant glucosinolate in the cabbage extract, meanwhile, gluconapin was identified in broccoli, cauliflower, and black radish. Neoglucobrassicin was the major glucosinolate found only in cauliflower. Methoxyglucobrassicin from broccoli, glucobrassicanapin, 4-hydroxyglucobrassicin, and glucoraphasatin from black radish completed the chromatographic profile of the extracts [19].

**Scheme 1:**
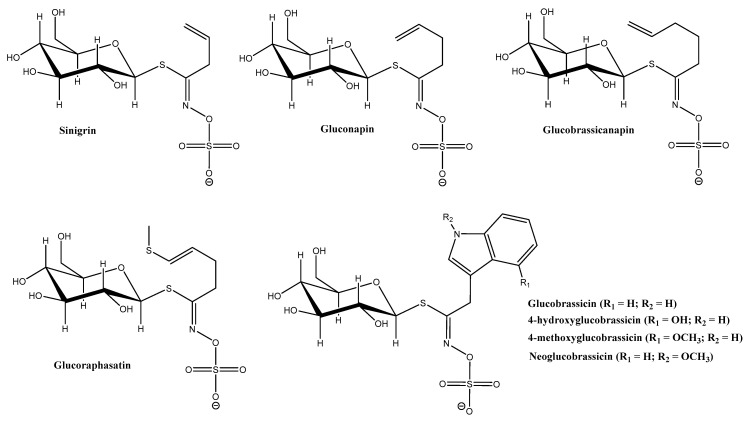
The structures of some major glucosinolates found in *Brassicaceae* plants.

The structures of the glucosinolates commonly found in *Brassicaceae* plants are shown in Scheme 1.

The highest antioxidant activity was obtained for cauliflower (9.54%) and broccoli (11.75%), while higher values of phenolic content were obtained for rapeseed (1713 mg GAE/L) and broccoli (1623 mg GAE/L).

The electrochemical behavior of the *Brassicaceae* extracts (extracts A and B) was evaluated. To achieve a corrosive environment, an aggressive support electrolyte (0.5 M H_2_SO_4_) was used.

Figure 3 reveals that the corrosion potential in the presence of all tested extracts shifted to noble values compared to blank. Moreover, both the cathodic and anodic current density present decreased values, meanwhile, Table 3 shows a change in *E_corr_* between 6–46 mV. This trend has been reported by other researchers [30,31]. An inhibitor is classified as an anodic-type or cathodic-type inhibitor when the change in *E*_corr_ is greater than 85 mV [30,31]. Also, a mixed-type inhibitor produces a reduction in both anodic and cathodic current densities [32].

It can be observed from Table 3 that the addition of *Brasicaceae* extracts leads to a significant decrease in the corrosion current densities (*I_corr_*) with a more pronounced drop for higher concentrations. The corrosion potential (*E_corr_*) values were only slightly affected by the presence of extracts and no explicit tendency in the change of *E_corr_* values depending on the extract concentration was observed. This type of behavior suggests that the extracts might act as pickling inhibitors [33]. This also indicates that the adsorption of the tested extracts on the steel surface leads to the blocking of the active sites slowing down corrosion. Considering the changes in the cathodic Tafel slope (*βc*) and anodic Tafel slope (*βa*), it can be concluded that the extracts’ actions are exerted on both anodic and cathodic reactions, thus resulting in a decrease in anodic dissolution and a delay of the cathodic hydrogen reaction [27,34]. These results suggest that all tested extracts act as mixed-type corrosion inhibitors.

Furthermore, in addition to the fact that *E_corr_* values have been slightly positively shifted, a more obvious decrease in the *βa* compared to the *βc* values in the presence of extracts can be noticed, which indicates a mixed-type inhibition behavior with predominant control of the anodic reaction [35].

The adsorption of organic compounds from the extracts at the active sites of the electrode surface leads to the delay of metallic dissolution and hydrogen evolution reaction. These results prove the capacity of the studied extracts to act as green corrosion inhibitors. The presence of extracts in the corrosive medium diminishes the corrosion rates concomitant with the shifting of the corrosion current density to lower values relative to the blank (Table 3).

The lowest corrosion rates, 1.98, 7.97, and 9.43 mm/year, were obtained for extracts A of broccoli, cauliflower, and black radish, respectively. This means that inhibitor efficiency increases with an increase in the inhibitor concentrations.

For plant extracts, both in anodic and cathodic domains, after a specific potential, the current-vs.-potential characteristics no longer change significantly. This behavior could be associated with the desorption of the adsorbed film of inhibitors on the surface of the electrode in acidic media. Above the desorption potential, the desorption rate of inhibitors is raised more than its adsorption [27].

The values obtained for the corrosion rates correlate with those calculated for inhibition efficiency (*IE*) and are presented in Table 3.

The values of inhibition efficiency obtained from the electrochemical measurements clearly increase with the concentration of the extracts and follow the order of broccoli > cauliflower > black radish > cabbage > rapeseed for extracts A. As for extracts B, the extract of broccoli can be found before rapeseed and the rest maintain the same trend. The inhibition efficiency for extracts A ranged between 89–99%. Only for rapeseed, an inhibition of 52.79% was obtained. All these results recommend the tested extracts as potent corrosion inhibitors.

Weight loss experiments (Table 4) were chosen to complete the results obtained by electrochemical tests for the determination of corrosion rates and inhibition efficiency. Although it is a time-consuming method, the benefit of this gravimetric method recommends its use. The advantages are based on the use of experimental conditions that more closely resemble real-life environments and the provided results that are likely to be more reliable [36].

All tested extracts proved to inhibit corrosion even if the exposure time was increased from minutes in the case of electrochemical tests to 24 h in the case of gravimetrical measurements, with inhibition efficiency values between 22.48 and 98.66%. However, except for broccoli extract, for which similar results were obtained by both tests (IE = 98–99%), the values of inhibition efficiency for the rest of the extracts, after 24 h of immersion in a corrosive media, decreased considerably. Thus, the cabbage extract lost 67% of its effectiveness, cauliflower 34%, black radish 17%, and rapeseed 58%. So, it seems that the difference in measurement times generally influences the inhibition capacity of the tested extracts. The weight loss experiments demonstrated the great potential of broccoli extract to inhibit the corrosion of the steel at 20 °C after a long period of immersion in an acidic solution.

The effect of temperature on the corrosion of steel in the presence and absence of *Brassicaceae* extracts was also monitored by weight loss measurements between 20–60 °C. The results presented in Table 4 reveal that the corrosion rate increases with the increase in the temperature, though to a lesser extent in the case of the presence of inhibitors. This behavior of the extracts in an acidic media is due to the increase in surface coverage by increasing the inhibitor concentration [28].

Temperature plays an important role in understanding the inhibitive mechanism of the corrosion process. To assess the temperature effect, the weight loss results (Table 4) were used to study the activation of the inhibition process by the means of Arrhenius Equation (4).

It is obvious that the apparent energy of activation increased in the presence of *Brassicaceae* extracts, compared to the uninhibited solution (Table 5). This increase suggests physical adsorption of the inhibitor on the steel surface. Also, the increase in the activation energy can be explained by an appreciable decrease in the adsorption of the inhibitor on the steel surface by an increase in the temperature. This decrease in adsorption leads to higher corrosion rates due to the increased exposed surface area of the steel towards the corrosive solution [28].

However, chemical adsorption of the inhibitor on a steel surface can also be involved. This kind of interaction involves charge sharing or charge transfer from the inhibitor to the atoms of the Fe in order to form a coordinate bond [28]. Moreover, the physical adsorption suggested by the trend of activation energy cannot be considered critical due to the competitive adsorption with water molecules, whose removal from the steel surface also requires some activation energy. Therefore, it can be considered that the adsorption of extracts’ compounds on the steel surface occurs through both physical and chemical phenomena, concurrently [37].

The values of the enthalpy of activation (ΔH*) and entropy of activation (ΔS*) for steel dissolution in 0.5 M H_2_SO_4_ in the presence and absence of inhibitor extracts (Table 5) were calculated using Equation (5). The positive values for ΔH* in the absence and presence of extracts reflect the endothermic nature of metal dissolution during the corrosion process [27,38,39]. In addition, the enthalpy values increase in the presence of the extracts compared to free the 0.5 M H_2_SO_4_ solution indicating a higher protection efficiency [28,40].

Regarding the entropy of activation (ΔS*), it can be seen that its values have increased in the presence of the inhibitor compared to the uninhibited solution. The gain in entropy suggests an increase in the disordering on going from the reactant to the metal–solution interface and can be attributed to the increase in solvent entropy and to more positive water desorption enthalpy [27].

Positive entropy of activation was obtained in the presence of broccoli extract while negative values but higher than ΔS* value for the free acid solution were obtained for the other tested extracts. Large and negative values of ΔS* indicate that the activation complex in the rate-determining step represents an association rather than a dissociation step, meaning that a decrease in disordering takes place on going from reactants to the activated complex [37,41].

The surface morphology of both steel electrodes from potentiodynamic polarization tests (Figure 4) and steel disks from weight-loss experiments (Figure 6) was examined using the SEM technique. The inhibition effect of the extracts can be clearly observed from the SEM images, especially for more concentrated extracts. The SEM images reveal that the surface was intensely injured in the absence of extracts. The surface damage was reduced in the presence of inhibitors, probably due to the protective film adsorbed on the steel surface that is responsible for the corrosion inhibition. The protective effect increased when using higher concentrated extracts. The aspect of the steel surface proved the presence of the shielding film adsorbed on it and is in agreement with the *IE* values presented in Table 3 and Table 4.

The results of the EDX analysis on the steel surface before and after the weight loss tests conducted at 20 °C are displayed in Figure 6 and Table 6. The EDX spectra of uncorroded steel shows the characteristics’ peaks of the elements constituting the steel sample (C, Si, P, S, Mn, Fe). The EDX spectra of the uninhibited steel disk (blank) show the normal peaks ascribed to general corrosion in sulfuric acid. As for inhibited solutions, the EDX spectra showed additional peaks characteristic of the existence of O and Cl, and differences in the weight percentage of the elements. These elements can originate from the compounds contained in the extracts. For example, we have demonstrated that the studied *Brassicaceae* extracts are rich in glucosinolates. These are sulfur- and nitrogen-containing glycosides, with a 2-hydroxymethyl-tetrahydro-pyran-3,4,5-triol moiety and a sulfide group, among other structures, which can contribute to the change in the weight percentage of elements such as C, O, and S on the steel surface. These results indicate that the inhibitor extracts adsorbed on the steel surface with different degrees of surface coverage, which can be correlated to their capacity to inhibit corrosion.

In order to elucidate the nature of the protective layer formed at the steel surface, the FT-IR spectra of the extracts and that of the steel surface after immersion in the inhibited and uninhibited solution of 0.5 M H_2_SO_4_ for 24h, at 20 °C, were evaluated. Generally, the FT-IR spectra of the films look almost similar to that of the corresponding extract. However, the intensity of some absorption bands of the steel surface decreased or their vibrations were shifted.

As shown in Table 7, the deviations are observed for functional groups from glucosinolates, so we can conclude that the glucosinolates were adsorbed on the steel surface as a protective anticorrosion film. A [Fe-extract functional groups]^2+^ complex is formed by covalent or coordinate bonds between nonbonding electrons in N, O, or C = C and vacant Fe d-orbital. It should be mentioned that plant extracts are complex matrices, so such complexes can be stable or soluble and consequently can act through a corrosion retarding or accelerating mechanism, concurrently. This can explain the extracts’ behaviors when increasing the temperature, i.e., the stable complex formation dominates at an increasing concentration until a critical concentration, where the formation of a soluble complex dominates [24].

## 5. Conclusions

This paper has demonstrated the anticorrosion properties of *Brassicaceae* extracts on steel materials in an acidic environment. Inhibition efficiency values increased with the increase in inhibitor concentration and decreased with an increase in temperature. The tested extracts act as mixed-type corrosion inhibitors, proving anticorrosion effects even at a low concentration. No correlation between antioxidant activities, total phenolic, and inhibition efficacy was observed. The mechanisms of corrosion inhibition consist of physical and chemical adsorption of glucosinolates on a steel surface concomitant with the formation of [Fe-extract functional groups]^2+^ complexes. Among the five *Brassicaceae* ethanolic extracts, broccoli extract was the best inhibitor for the corrosion of steel in a 0.5 M H_2_SO_4_ solution. Given the behaviors of the *Brassicaceae* extracts, these can successfully substitute for the conventional toxic inhibitors and could be used as green corrosion inhibitors.

## Figures and Tables

**Table 1:** Antioxidant activities and total phenolic content of extracts A.

Extracts	Antioxidant Activity, Remaining DPPH, %	Total Phenolics, mg GAE/L
Broccoli	11.75	1623.4 ± 25.1
Cabbage	15.79	1008.8 ± 9.6
Black radish	22.03	1313.5 ± 16.4
Rapeseed	15.13	1713.0 ± 42.8
Cauliflower	9.54	1380.7 ± 19.4

**Table 2:** The steel composition of the working electrode.

Element	C	Si	Mn	P	S	Fe
Percentage, wt.%	0.12–0.15	0.10–0.35	0.70–1.10	0.03	0.07–0.13	98.21–98.98

**Table 3:** Potentiodynamic polarization parameters.

Sample	*E_corr_*, mV	*Rp*, Ohm·cm^2^	*I_corr_*, mA/cm^2^	*βa*, mV	*βc*, mV	*v_corr_*, mm/year	*IE*, %
0.5 M H_2_SO_4_	−454.7	3.20	10.13	189.6	−202.2	118.5	-
Broccoli A	−408.9	84.60	0.17	82.6	−83.8	1.98	98.33
Broccoli B	−435.4	6.83	4.83	154.7	−187.6	56.54	52.30
Cabbage A	−436.6	24.54	1.05	95.2	−141.3	12.26	89.65
Cabbage B	−435.3	7.39	3.26	130.8	−145.0	38.14	67.82
Cauliflower A	−433.9	25.73	0.68	84.1	−108.2	7.97	93.27
Cauliflower B	−430.4	15.16	1.28	90.8	−116.4	14.99	87.35
Black radish A	−430.8	23.80	0.81	108.0	−110.8	9.43	92.05
Black radish B	−434.9	8.84	2.74	108.9	−146.5	32.02	72.98
Rapeseed A	−448.6	6.95	4.78	152.5	−184.2	55.96	52.79
Rapeseed B	−442.5	4.87	7.39	186.6	−212.3	86.38	27.13

**Table 4:** Weight loss results of steel in 0.5 M H_2_SO_4_, at different temperatures, in the absence and presence of *Brassicaceae* extracts A.

Sample	v_corr_ (mm/Year)				IE (%)			
	20 °C	40 °C	50 °C	60 °C	20 °C	40 °C	50 °C	60 °C
0.5 M H_2_SO_4_	58.66	85.70	90.41	91.69	-	-	-	-
Broccoli	0.78	33.67	56.50	79.57	98.66	60.71	37.51	13.22
Cabbage	40.98	67.41	83.12	89.57	30.13	21.35	8.06	2.32
Cauliflower	22.31	63.82	78.55	89.19	61.96	25.53	13.11	2.73
Black radish	13.55	61.59	74.13	89.31	76.90	28.14	18.00	2.60
Rapeseed	45.47	75.33	86.37	89.71	22.48	12.10	4.46	2.16

**Table 5:** Activation parameters for steel in 0.5 M H_2_SO_4_ in the absence and presence of *Brassicaceae* extracts A.

Inhibitor	Linear Regression Equation (4)	R^2^ Equation (4)	Ea (kJ mol^−1^)	Linear Regression Equation (5)	R^2^ Equation (5)	ΔH* (kJ mol^−1^)	ΔS* (J mol^−1^ K^−1^)
H_2_SO_4_	y = −0.495x + 3.476	0.893	9.47	y = −0.828x + 1.260	0.811	6.88	−187.06
Broccoli	y = −5.055x + 17.319	0.914	96.77	y = −11.328x + 33.137	0.910	94.18	77.96
Cabbage	y = −0.861x + 4.562	0.978	16.48	y = −1.671x + 3.761	0.968	13.89	−166.27
Cauliflower	y = −1.513x + 6.555	0.942	28.97	y = −3.173x + 8.352	0.930	26.38	−128.10
Black radish	y = −2.047x + 8.186	0.921	39.19	y = −4.403x + 12.108	0.910	36.60	−96.88
Rapeseed	y = −0.752x + 4.245	0.943	14.40	y = −1.421x + 3.031	0.916	11.81	−172.34

**Table 6:** Percentage of atomic contents of elements obtained from EDX spectra.

Elements	Atomic content (%)						
	Steel Disks	H_2_SO_4_	Broccoli	Cabbage	Cauliflower	Black radish	Rapeseed
C	0.22	-	19.61	-	17.24	18.92	2.45
Si	0.07	0.35	2.64	0.30	1.70	0.68	1.03
P	0.02	0.59	0.44	0.30	0.30	0.37	0.34
S	0.09	1.42	0.65	11.22	0.63	0.68	2.67
Mn	1.04	0.63	-	0.34	0.78	0.68	56.46
Fe	98.56	57.35	60.99	29.63	57.00	46.47	37.05
O	-	39.52	15.67	58.07	22.00	31.55	-
Cl	-	0.15	-	0.14	0.34	0.66	-

**Table 7:** The changes in the FT-IR spectra of inhibitor film compared to *Brassicaceae* extracts.

Brassicaceae Extracts	Inhibitor Film
3284 cm^−1^ OH stretching, intermolecular bonded, intense, large	Decreased intensity or shifted to 3330 cm^−1^
1661 cm^−1^ C = N stretching, medium	Decreased intensity, absent or shifted to 1644 cm^−1^
1625 cm^−1^ C = C stretching, medium	Absent
1392, 1337 cm^−1^ OH bending, intense	Shifted to 1416 cm^−1^
1029 cm^−1^ C-O stretching, intense	Decreased intensity, shifted to 1115 cm^−1^

## Data Availability

Not applicable.
